# Molecular Phylogeny and Historical Biogeography of *Goodyera* R. Br. (Orchidaceae): A Case of the Vicariance Between East Asia and North America

**DOI:** 10.3389/fpls.2022.850170

**Published:** 2022-05-02

**Authors:** Tae-Hee Kim, Joo-Hwan Kim

**Affiliations:** Department of Life Science, Gachon University, Seongnam, South Korea

**Keywords:** next-generation sequencing, plastid genome, phylogenomic, age estimation, Bering Land Bridge

## Abstract

Understanding of intercontinental distribution in the Northern Hemisphere has attracted a lot of attention from botanists. However, although Orchidaceae is the largest group of angiosperms, biogeographical studies on the disjunctive pattern have not been sufficient for this family. *Goodyera* R. Br. (tribe Cranichideae, subfamily Orchidoideae, family Orchidaceae) is widely distributed in temperate and tropical regions. Although the phylogenetic relationship of *Goodyera* inferred from both morphological and molecular data has been conducted, the sampled taxa were mainly distributed in Asia regions that resulted in non-monophyly of this genus. In this study, the complete plastid genomes of *Goodyera*, generated by next-generation sequencing (NGS) technique and sampled in East Asia and North America, were used to reconstruct phylogeny and explore the historical biogeography. A total of 18 *Goodyera* species including seven newly sequenced species were analyzed. Based on 79 protein-coding genes, the phylogenetic analysis revealed that *Goodyera* could be subdivided into four subclades with high support values. The polyphyletic relationships among *Goodyera* taxa were confirmed, and the unclear position of *G. foliosa* was also resolved. The datasets that are composed of the 14 coding sequences (CDS) (*mat*K, *atp*F, *ndh*K, *acc*D, *cem*A, *clp*P, *rpo*A, *rpl*22, *ndh*F, *ccs*A, *ndh*D, *ndh*I, *ndh*A, and *ycf* 1) showed the same topology derived from 79 protein-coding genes. Molecular dating analyses revealed the origin of *Goodyera* in the mid-Miocene (15.75 Mya). Nearctic clade of *Goodyera* was diverged at 10.88 Mya from their most recent common ancestor (MRCA). The biogeographical reconstruction suggests that subtropical or tropical Asia is the origin of *Goodyera* and it has subsequently spread to temperate Asia during the Miocene. In addition, Nearctic clade is derived from East Asian species through Bering Land Bridge (BLB) during the Miocene. The speciation of *Goodyera* is most likely to have occurred during Miocene, and climatic and geological changes are thought to have had a part in this diversification. Our findings propose both origin and vicariance events of *Goodyera* for the first time and add an example for the biogeographical history of the Northern Hemisphere.

## Introduction

The intercontinental distribution of plants has been the subject of many botanist's research to date. Since Gray (1846) has investigated the vicariance of plants in Northern Hemisphere, many studies of vicariance and disjunction between East Asia and North America have been conducted till date (Donoghue et al., 2001; Wen et al., 2010; Kim et al., 2017, 2019). According to the studies, occasions such as continental drift and global climatic changes may have caused the fragmentation of the Northern Hemisphere. Wen et al. (2010) found that most temperate lineages had a dominance of directionality from East Asia to North America by identifying 98 lineages with disjunct distribution between two regions. Even though the Orchidaceae is the largest family among angiosperms, the results showed that only one lineage is in the orchid family. Currently, biogeographical analyses have been carried out on orchids (Guo et al., 2012; Givnish et al., 2016; Smidt et al., 2021), but the comprehension of the disjunctive pattern of orchids is still lacking compared to the other groups.

The genus *Goodyera* R. Br., commonly known as ladies' tresses or jewel orchids, is the widespread genus of the subtribe Goodyerinae (tribe Cranichideae; subfamily Orchidoideae; family Orchidaceae). It consists of about 99 species and is distributed in Asia, North America (including Mexico), Europe, Southern Africa, northeast Australia, Madagascar, and the Pacific Islands; ca. 25 species in Indomalaya realm, 30 species in Palearctic realm, 17 species in New World (mostly Nearctic realm and Mexico), and 15 species in Australasian realm (Chen et al., 2009; Chase et al., 2015; Hu et al., 2016; POWO, 2019). Many species of *Goodyera* are terrestrial (rarely epiphytic), which grow on moist grounds or mossy rocks of mountains and have characteristics of leaves with white or golden reticulate veins, elongate and creeping rhizome, hoods including dorsal sepal and petals, resupinate flowers, and concave-saccate labellum (Chen et al., 2009; Hu et al., 2016; Liu et al., 2019).

According to Schlechter (1911), *Goodyera* was divided into two sections: *Otosepalum* and *Goodyera*, which were recognized by lateral sepals reflexed and later sepals normally spreading, respectively. The treatment of Schlechter (1911) was followed by many scholars (Seidenfaden, 1978; Seidenfaden et al., 1992; Pearce and Cribb, 2002). On the other hand, different characteristics were found as taxonomic keys such as reticulate venation or marking on the surface of the leaves, rosulate leaves or not, etc. (Lang, 1999; Chen et al., 2009; Hu et al., 2016). Previously, a phylogeny study based on the nuclear ribosomal internal transcribed spacer (ITS) region revealed the monophyly of Korean *Goodyera* species (Shin et al., 2002). Juswara (2010) used the ITS and two chloroplast loci (*trn*L-F and *rpl*16) to clarify the phylogenetic relationships of several *Goodyera* species and demonstrated the polyphyly of *Goodyera*. Smidt et al. (2020) compared complete chloroplast sequences and concluded that *Goodyera* is biphyletic, although the clade was supported with a high value. Although different studies have been conducted, the monophyly of *Goodyera* in East Asia and North America has not been clarified.

Hu et al. (2016) indicated that *Goodyera* is divided into four sections with specific distribution: section *Goodyera* in the Northern Hemisphere, section *Otosepalaum* in Indomalaya and Australasian realm, section *Reticulum* in Indomalaya and Palearctic realm, and a new section composed by *G. procera* in the Indomalaya realm, a result of molecular data including ITS, *trn*L-F, and *mat*K. This paper only identified these geographical distributions but does not conduct a biogeographical analysis or explanation. Furthermore, the disjunction patterns of *Goodyera* between East Asia and North America have received the least attention.

In this study, we aim to (1) carry out the plastid genome evolution of *Goodyera* in terms of sequence variation, and gene content and order inferred from seven newly sequenced genomes and the available sequences on National Center for Biotechnology Information (NCBI); (2) reconstruct the phylogenetic relationships of the genus *Goodyera* based on 79 protein-coding genes data; (3) conduct nucleotide diversity to find phylogenetically valuable genes; and (4) investigate the origin and divergence periods of *Goodyera* species between East Asia and North America using biogeographical analysis.

## Materials and Methods

### Taxon Sampling and DNA Extraction

Fresh leaf of *Goodyera* was collected in the field in South Korea or North America and dried directly with silica gel at room temperature until conducting DNA extraction (Supplementary Table S1). In total, 18 *Goodyera* species (including seven new plastid genomes) and 11 additional related genera of 13 species from GenBank (except for *Spiranthes sinensis*, of which the plastid genome was sequenced in this study) were used. Voucher specimens were made for all used samples and deposited in the Gachon University Herbarium (GCU) with specific accession numbers. Total extracted DNAs of each sample were extracted using the modified 2X CTAB buffer method (Doyle and Doyle, 1987), checked by 1% agarose gel electrophoresis with ethidium bromide staining and measured the concentration by spectrophotometer (Biospec-nano; Shimadzu).

### Genome Sequencing, Assembly, and Annotation

The samples were sequenced using the Illumina MiSeq sequencing system (Illumina, Seoul, Korea) (Supplementary Table S1). The resulting next-generation sequencing (NGS) raw reads from MiSeq were imported and trimmed with a 2% error probability limitation to remove poor quality reads by Geneious 7.1.9 (Kearse et al., 2012). Plastid genome of *Goodyera fumata* (GenBank accession no. = NC_026773.1) was used as a reference sequence to perform “map to reference” using Geneious 7.1.9 to the isolation of cpDNA reads (Kearse et al., 2012). *De novo* assembly was implemented to reassemble reads using Geneious 7.1.9 (Kearse et al., 2012). Contigs from the *de novo* assembly were used as the reference sequences to reassemble raw reads repeatedly until complete the plastome structures were obtained. Gene content and order were annotated using *Goodyera fumata* (GenBank accession no. = NC_026773.1) as a reference using 80% similarity to identify genes in Geneious, and all transfer RNAs (tRNAs) were confirmed by tRNAscan-SE with default search mode (Chan and Lowe, 2019). Complete circular plastome map was illustrated using OGDraw (Greiner et al., 2019).

### Comparative Genome Analysis

Genome structure, size, and gene contents of 18 *Goodyera* complete plastid genomes were compared. The GC content was calculated and compared using Geneious. The whole plastid genome sequences of *Goodyera* species were aligned and compared using mVISTA program in the Shuffle-LAGAN mode (Brudno et al., 2003; Frazer et al., 2004). For the mVISTA plot, we used the annotated cpDNA of *Anoectochilus emeiensis* as a reference. To find the phylogenetically informative genes, the sliding window analysis using DnaSP v. 6.0 program was used to examine the nucleotide diversity (Pi) of plastid protein-coding genes, transfer RNA genes, and ribosomal RNA genes among the 18 *Goodyera* species (Rozas et al., 2017). For the sequence divergence analysis, we applied the window size of 100 bp with a 25-bp step size (Supplementary Table S2). The inverted repeat (IR) and single copy (SC) boundaries of the 18 *Goodyera* species were compared and illustrated using IRscope (Amiryousefi et al., 2018).

### Phylogenomic Analysis

A total of 31 plastid genome sequences were subjected to the phylogenetic study (Table 1). Among them, 23 plastid genome was obtained from NCBI, of which *Ophrys sphegodes* (GenBank accession no. = AP018717.1) and *Habenaria linearifolia* (GenBank accession no. = MT863538.1) were used as outgroups (Table 1). For phylogenomic analyses, 79 protein-coding genes were extracted and aligned using the MUSCLE program of Geneious 7.1.9 (Kearse et al., 2012). Gaps were treated as missing data. We performed maximum parsimony (MP), maximum likelihood (ML), and Bayesian inference (BI) based on the combined sequences of 79 protein-coding genes to reconstruct the relationships of *Goodyera* and related taxa. The MP analyses were conducted in PAUP^*^ v4.0a (Swofford, 2003). All characters equally weighted and unordered. The searches of 1,000 random taxon addition replicates used tree-bisection-reconnection (TBR) branch swapping, and MulTrees permitted ten trees to be held at each step. Bootstrap analyses (PBP, parsimony bootstrap percentages, 1,000 pseudoreplicates) were conducted to examine internal support for individual clades with the same parameters.

**Table 1:** Comparison of the plastome features of *Goodyera* and related taxa.

**Taxa**	**Subtribe**	**Tribe**	**Subfamily**	**Length (bp) and G+C content (%)**				**GenBank accession number**
				**LSC**	**SSC**	**IR**	**Total**	
*Goodyera biflora*	Goodyerinae	Cranichideae	Orchidoideae	83,466 (34.9%)	17,893 (29.9%)	26,508 (43.3%)	154,375 (37.2%)	OM314910
*Goodyera foliosa* ^*^	Goodyerinae	Cranichideae	Orchidoideae	83,442 (35.1%)	18,126 (30.0%)	26,273 (43.4%)	154,114 (37.3%)	MN443774
*Goodyera fumata* ^*^	Goodyerinae	Cranichideae	Orchidoideae	84,077 (35.1%)	18,342 (29.9%)	26,612 (43.3%)	155,643 (37.3%)	KJ501999
*Goodyera henryi*	Goodyerinae	Cranichideae	Orchidoideae	83,596 (35.1%)	17,720 (30.3%)	26,488 (43.4%)	154,292 (37.4%)	OM314911
*Goodyera malipoenesis* ^*^	Goodyerinae	Cranichideae	Orchidoideae	83,273 (34.9%)	18,084 (29.8%)	26,665 (43.3%)	154,687 (37.2%)	MW589514
*Goodyera marginata* ^*^	Goodyerinae	Cranichideae	Orchidoideae	81,834 (34.9%)	17,970 (29.7%)	26,438 (43.2%)	152,680 (37.2%)	MW589515
*Goodyera nankoensis* ^*^	Goodyerinae	Cranichideae	Orchidoideae	81,775 (34.9%)	17,945 (29.6%)	26,423 (43.2%)	152,566 (37.1%)	MW589516
*Goodyera procera* ^*^	Goodyerinae	Cranichideae	Orchidoideae	82,766 (35.3%)	18,170 (30.2%)	26,553 (43.4%)	154,042 (37.5%)	MW589517
*Goodyera pubescens*	Goodyerinae	Cranichideae	Orchidoideae	82,101 (34.9%)	17,876 (29.9%)	26,220 (43.2%)	152,417 (37.2%)	OM314912
*Goodyera repens* ^*^	Goodyerinae	Cranichideae	Orchidoideae	82,101 (34.9%)	17,983 (29.6%)	26,227 (43.3%)	152,538 (37.2%)	MW589518
*Goodyera rosulacea* ^*^	Goodyerinae	Cranichideae	Orchidoideae	82,041 (34.5%)	17,720 (29.0%)	26,535 (42.9%)	152,831 (36.8%)	MN200390
*Goodyera schlechtendaliana*1	Goodyerinae	Cranichideae	Orchidoideae	82,741 (34.9%)	18,048 (29.7%)	26,535 (43.3%)	153,859 (37.2%)	OM314913
*Goodyera schlechtendaliana*2	Goodyerinae	Cranichideae	Orchidoideae	82,674 (34.8%)	17,999 (29.7%)	26,535 (43.3%)	153,743 (37.2%)	OM314914
*Goodyera seikoomontana* ^*^	Goodyerinae	Cranichideae	Orchidoideae	82,865 (34.9%)	18,236 (29.6%)	25,828 (43.2%)	152,757 (37.1%)	MW589520
*Goodyera striata*	Goodyerinae	Cranichideae	Orchidoideae	82,081 (34.8%)	17,871 (29.7%)	26,395 (43.3%)	152,742 (37.1%)	OM314915
*Goodyera velutina*	Goodyerinae	Cranichideae	Orchidoideae	83,523 (35.0%)	18,160 (30.0%)	26,157 (43.4%)	153,997 (37.3%)	OM314916
*Goodyera viridiflora* ^*^	Goodyerinae	Cranichideae	Orchidoideae	82,786 (34.8%)	18,331 (29.4%)	26,398 (43.3%)	153,913 (37.1%)	MW589521
*Goodyera yangmeishanensis* ^*^	Goodyerinae	Cranichideae	Orchidoideae	82,923 (34.9%)	17,871 (29.8%)	26,566 (43.3%)	153,926 (37.2%)	MW589522
*Aspidogyne longicornu* ^*^	Goodyerinae	Cranichideae	Orchidoideae	82,437 (34.5%)	18,015 (29.6%)	26,492 (43.1%)	153,436 (36.9%)	MN597437
*Anoectochilus emeiensis* ^*^	Goodyerinae	Cranichideae	Orchidoideae	82,670 (34.5%)	17,342 (29.3%)	26,319 (43.2%)	152,650 (36.9%)	LC057212
*Anoectochilus zhejiangensis* ^*^	Goodyerinae	Cranichideae	Orchidoideae	82,660 (34.4%)	17,201 (29.4%)	26,324 (43.2%)	152,509 (36.9%)	MW173020
*Cheirostylis chinensis* ^*^	Goodyerinae	Cranichideae	Orchidoideae	81,081 (34.3%)	14,769 (29.0%)	25,684 (42.9%)	147,218 (36.8%)	MN641483
*Erythrodes blumei* ^*^	Goodyerinae	Cranichideae	Orchidoideae	83,588 (34.9%)	18,140 (29.6%)	26,569 (43.3%)	154,866 (37.1%)	MW589509
*Ludisia discolor* ^*^	Goodyerinae	Cranichideae	Orchidoideae	82,675 (34.6%)	17,233 (29.7%)	26,573 (43.0%)	153,054 (37.0%)	KU578274
*Cyclopogon longibracteatus* ^*^	Spiranthinae	Cranichideae	Orchidoideae	83,704 (34.4%)	17,779 (29.6%)	26,506 (43.1%)	154,495 (36.9%)	MN597436
*Sauroglossum elatum* ^*^	Spiranthinae	Cranichideae	Orchidoideae	83,822 (34.5%)	17,734 (29.9%)	26,503 (43.2%)	154,562 (37.0%)	MN597432
*Spiranthes sinensis* ^*^	Spiranthinae	Cranichideae	Orchidoideae	83,446 (33.6%)	17,938 (28.5%)	25,701 (43.1%)	152,786 (36.2%)	MK936427
*Spiranthes sinensis*	Spiranthinae	Cranichideae	Orchidoideae	84,216 (33.6%)	18,266 (28.3%)	25,736 (43.1%)	153,954 (36.1%)	OM314917
*Prescottia stachyodes* ^*^	Cranichidinae	Cranichideae	Orchidoideae	83,373 (34.2%)	17,685 (29.4%)	26,493 (43.1%)	154,044 (36.7%)	MN597433
*Habenaria linearifolia* ^*^	Orchidinae	Orchideae	Orchidoideae	85,053 (34.2%)	17,680 (29.0%)	26,460 (43.0%)	155,653 (36.6%)	MT863538
*Ophrys sphegodes* ^*^	Orchidinae	Orchideae	Orchidoideae	80,471 (34.3%)	16,177 (29.3%)	25,053 (43.5%)	146,754 (36.9%)	AP018717

* *From NCBI*.

Before carrying out the ML and BI analyses, we used jModelTest version 2.1.7 to find the best model with Akaike's information criterion (AIC) (Guindon and Gascuel, 2003; Darriba et al., 2012). The best model for the combined dataset was the GTR+I+G. To carry out the ML searches, the IQ-TREE web server (http://iqtree.cibiv.univie.ac.at/) was used (Trifinopoulos et al., 2016). Support value (MBP, mean bootstrap percentage) was calculated with 1,000 replicates of ultrafast bootstrap (Stamatakis et al., 2008). We used MrBayes v3.2.6 for BI analyses (Ronquist et al., 2012). A total of two simultaneous runs were performed starting from random trees for at least 1,000,000 generations. Then, one tree was sampled for every 1,000 generations. In total, 25% of trees were discarded as burn-in samples. The remaining trees were used to construct a 50% majority-rule consensus tree, with the proportion bifurcations found in this consensus tree given as posterior probability (PP) to estimate the robustness of half of the BI tree. The effective sample size values were then checked for model parameters (at least 200). The phylogenetic trees were modified using FigTree v1.4.4 (Rambaut, 2018).

### Molecular Dating Analysis

Based on the phylogenetically useful chloroplast DNA (cpDNA)-coding genes identified by analyzing nucleotide diversity, we used BEAST v.1.8.3 (Drummond and Rambaut, 2007) to estimate the divergence times of *Goodyera*. The BEAUti interface was used to generate input files for BEAST, in which the GTR+I+R model, Yule speciation tree prior, and uncorrelated lognormal molecular clock model were applied. The Markov chain Monte Carlo (MCMC) analysis was 100 million generations, sampling parameters for every 1,000 generations. After discarding the first 10,000 (10%) trees as burn-in, the samples were summarized in a maximum clade credibility tree in TreeAnnotator v.1.8.3 (Drummond and Rambaut, 2007) using a PP limit of 0.50 and summarizing the mean node heights. The means and 95% higher posterior densities (HPDs) of age estimates were obtained from the combined outputs using tracer. The results were visualized using Figtree v.1.4.4. (Rambaut, 2018).

Age calibration was constrained to the phylogeny of *Goodyera* and its close relatives. Ramírez et al. (2007) published a pollen fossil dated to 15–20 Mya, *Meliorchis caribea* S.R.Ramírez, Gravend., R.B.Singer., C.R.Marshall, and N.E.Pierce and resolved its phylogenetic position within the extant subtribe Goodyerinae (subfamily Orchidoideae) based on its morphology. This fossil was used for the calibration point of Goodyerinae with a uniform prior distribution; a lower bound of 15 Mya and an upper bound of 20 Mya (Ramírez et al., 2007; Gustafsson et al., 2010; Iles et al., 2015). To improve the accuracy of calibration, we used an additional secondary calibration point; the crown age of Cranichideae, 42.82 Mya with normal prior distribution (mean = 42, SD = 3.5) following Smidt et al. (2020), from Givnish et al. (2015), who estimated the divergence time of all orchid subfamilies using concatenated nucleotide sequences of three plastid-coding genes (*atp*B, *psa*B, and *rbc*L).

### Ancestral Area Reconstruction

The biogeographical data for the Goodyerinae including *Goodyera* taxa were collected from herbarium specimens and the database (http://www.plantsoftheworldonline.org/, accessed on 20 July 2021) of the Plants of the World Online (POWO, 2019). The distribution range of Goodyerinae was divided into five regions, consisting of (A) Southeast Asia, (B) East Asia, (C) South Asia, (D) North America, and (E) South America. Each species was coded based on the entire range of the species due to the ambiguity of the biogeographical source for the sample from NCBI. The ancestral area reconstruction and the estimation of the spatial patterns of geographic diversification within *Goodyera* were inferred using the Bayesian binary method (BBM) as implemented in Reconstruct Ancestral State in Phylogenies (RASP v3.1) (Yu et al., 2015). BBM was selected due to its characteristics, which suggests single-distribution areas for ancestral nodes than others and provides the deliberate and instructive results (Müller et al., 2015; Ito et al., 2017; Ha et al., 2018).

For the BBM analysis, we used all post-burn-in trees obtained from the BEAST analysis. The BBM was run using the fixed state frequencies model (Jukes-Cantor) with equal among-site rate variation for 50,000 generations, 10 chains each, and two parallel runs. The consensus tree used to map the ancestral distribution of each node was obtained with the Compute Condense option in RASP from stored trees. The maximum number of ancestral areas was set to five.

## Results

### Comparative Plastid Genome Structure and Nucleotide Diversity

**Figure 1:**
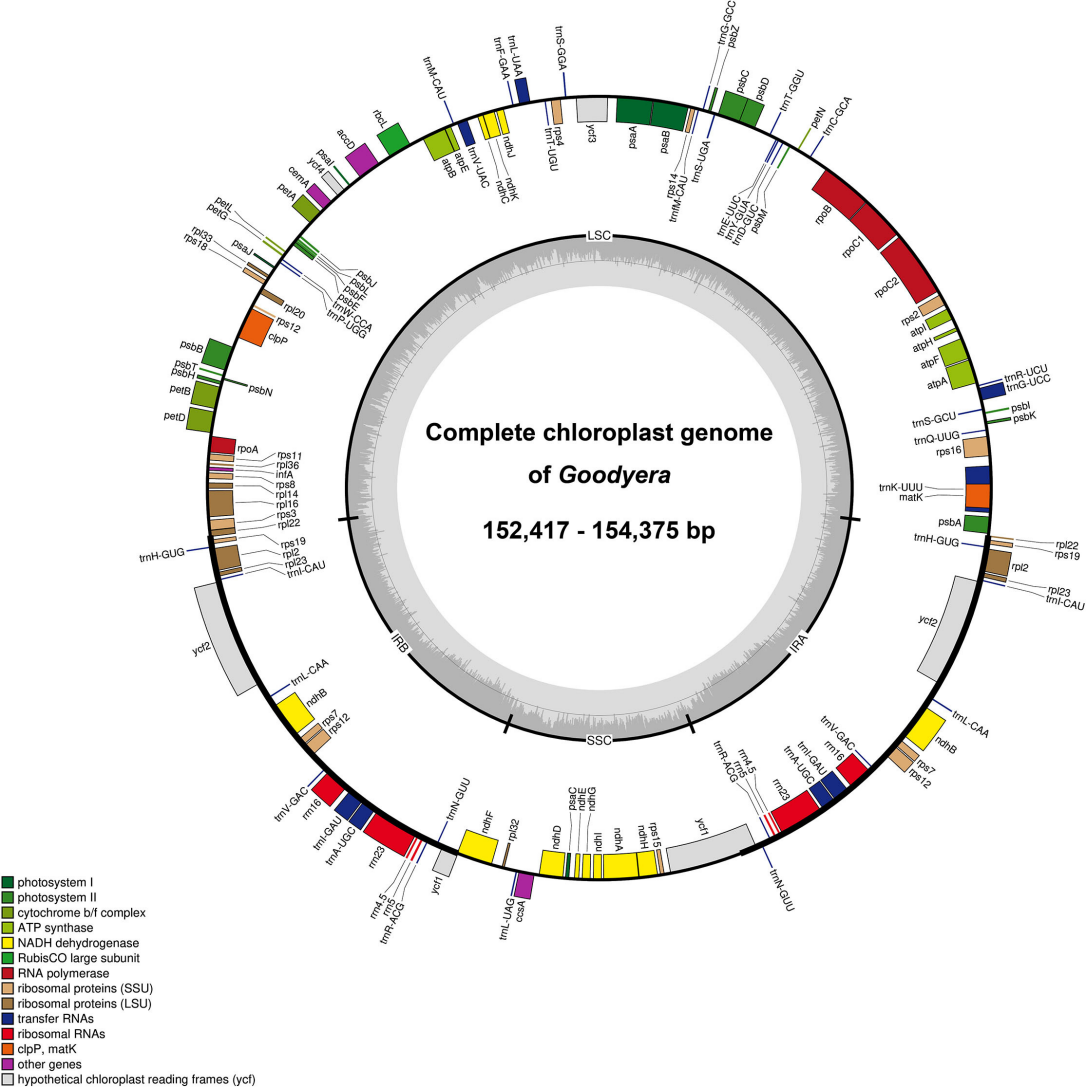
Representative plastid genome of *Goodyera*. The colored boxes represent functional groups of plastid-coding genes. Genes shown inside the circle are transcribed clockwise, whereas genes shown outside the circle are transcribed counter-clockwise. The small gray bar graphs in the inner circle show the GC contents.

The seven newly sequenced plastomes of *Goodyera* ranged from 152,417 to 154,375 bp with the typical quadripartite structure (Figure 1 and Supplementary Figure S1). Plastid genome of 18 *Goodyera* species varied from 152 kb (*G. pubescens*) to 155 kb (*G. fumata*) in length with 37.2% of average GC content (Table 1). In *Goodyera, G. procera* has the highest GC content (37.5%) and *G. rosulacea* has the lowest GC content (36.8%) (Table 1). Plastid genomes of *Goodyera* encoded 117 unique genes including 79 protein-coding genes (CDS), 30 tRNA genes, and four ribosomal RNA (rRNA) genes (Table 2). A number of four species (*G. nankoensis, G. marginata, G. repens*, and *G. rosulacea*) have a pseudo-*ndh*B due to point mutation.

**Table 2:** Gene composition within plastid genomes of *Goodyera* species.

**Groups of genes**		**Names of genes**	**No**.
RNA genes	Ribosomal RNAs	*rrn*4.5^x2^, *rrn*5^x2^, *rrn*16^x2^, *rrn*23^x2^	8
	Transfer RNAs	*trn*K-UUU^a^, *trn*Q-UUG, *trn*S-GCU, *trn*G-UCC^a^, *trn*R-UCU, *trn*C-GCA, *trn*D-GUC, *trn*Y-GUA, *trn*E-UUC, *trn*T-GGU, *trn*S-UGA, *trn*G-GCC, *trn*fM-CAU, *trn*S-GGA, *trn*T-UGU, *trn*L-UAA^a^, *trn*F-GAA, *trn*V-UAC^a^, *trn*M-CAU, *trn*L-UAG, *trn*W-CCA, *trn*P-UGG, *trn*H-GUG^x2^, *trn*I-CAU^x2^, *trn*L-CAA^x2^, *trn*V-GAC^x2^, *trn*I-GAU^a^^x2^, *trn*A-UGC^a^^x2^, *trn*R-ACG^x2^, *trn*N-GUU^x2^	38
Protein genes	Photosystem I	*psa*A, *psa*B, *psa*C, *psa*I, *psa*J	5
	Photosystem II	*psb*A, *psb*B, *psb*C, *psb*D, *psb*E, *psb*F, *psb*H, *psb*I, *psb*J, *psb*K, *psb*L, *psb*M, *psb*N, *psb*T, *psb*Z	15
	Cytochrome	*pet*A, *pet*B^a^, *pet*D^a^, *pet*G, *pet*L, *pet*N	6
	ATP synthases	*atp*A, *atp*B, *atp*E, *atp*F^a^, *atp*H, *atp*I	6
	Large unit of Rubisco	*rbc*L	1
	NADH dehydrogenase	*ndh*A^a^, *ndh*B^a^^x2^^(Ψ)^, *ndh*C, *ndh*D, *ndh*E, *ndh*F, *ndh*G, *ndh*H, *ndh*I, *ndh*J, *ndh*K	12
	ATP-dependent protease subunit P	*clp*P^b^	1
	Envelope membrane protein	*cem*A	1
Ribosomal proteins	Large units of ribosome	*rpl*2^a^^x2^, *rpl*14, *rpl*16^a^, *rpl*20, *rpl*22, *rpl*23^x2^, *rpl*32, *rpl*33, *rpl*36	11
	Small units of ribosome	*rps*2, *rps*3, *rps*4, *rps*7^x2^, *rps*8, *rps*11, *rps*12^b^^x2^, *rps*14, *rps*15, *rps*16^a^, *rps*18, *rps*19^x2^	15
Transcription/ translation	RNA polymerase	*rpo*A, *rpo*B, *rpo*C1^a^, *rpo*C2	4
	Initiation factor	*inf*A	1
	Miscellaneous protein	*acc*D, *ccs*A, *mat*K	3
	Hypothetical proteins and conserved reading frames	*ycf*1, *ycf*2^x2^, *ycf*3^b^, *ycf*4	5
Total			132

a *Gene with one intron*.

b *Gene with two introns*.

x2 *Duplicated gene*.

Ψ *Pseudogene in G. nankoensis, G. marginata, G. repens, and G. rosulacea*.

**Figure 2:**
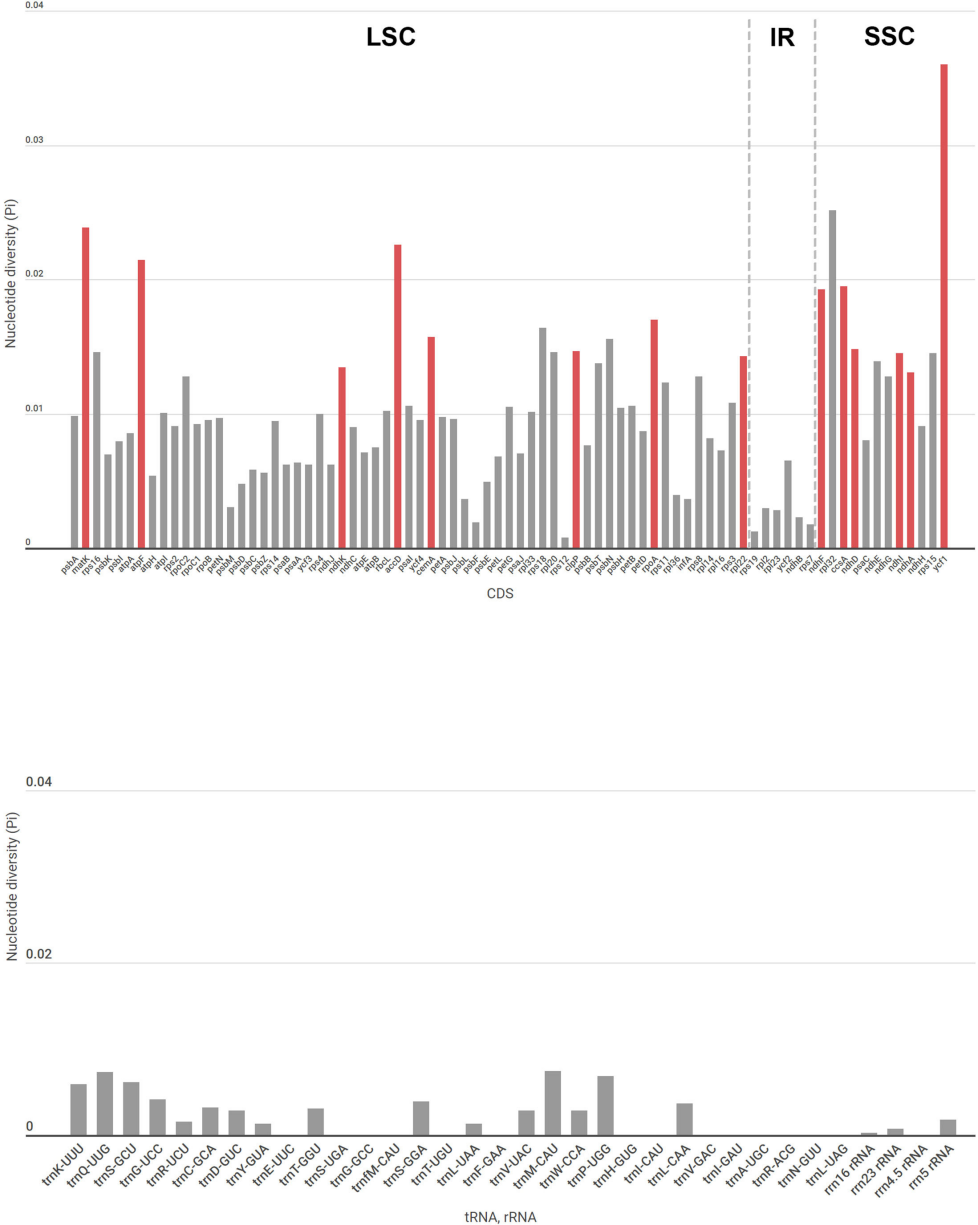
Nucleotide diversity (Pi) values in protein-coding genes, tRNA, and rRNA in 18 *Goodyera* species. The dashed lines are the borders of the LSC, IR, and SSC regions. The red bars indicate phylogenetically informative genes with Pi > 0.013 and over 500 bp length in our study.

Unlike other *Goodyera* species, *G. schlechtendaliana* has an inversion (about 1.0 kb) including *psb*K in the large single copy (LSC) region (Supplementary Figure S2). In comparison with *Goodyera* species, *Aspidogyne longicornu* and *Erythrodes blumei* do not have remarkable differences in sequence variability of the whole plastid genome. The boundaries of the quadripartite structure of the plastid genome were similar among *Goodyera* species (Supplementary Figure S3). To identify features among the 18 *Goodyera* plastid genomes, nucleotide divergences of CDS, tRNA, and rRNA were analyzed (Figure 2). In CDS, nucleotide diversity (Pi) has an average of 0.01029. The highest value was 0.03599 (*ycf* 1), followed by 0.02514 (*rpl*32) and 0.02387 (*mat*K), whereas the lowest value was 0.00079 (*rps*12). RNAs have an average of 0.00196 with the Pi values ranged from 0 to 0.00735 (*trn*Q-UUG). By selecting the datasets of CDS (*mat*K, *atp*F, *ndh*K, *acc*D, *cem*A, *clp*P, *rpo*A, *rpl*22, *ndh*F, *ccs*A, *ndh*D, *ndh*I, *ndh*A, and *ycf* 1) with high values (Pi > 0.013) and over 500 bp in length, we established the phylogenetically useful genes for the *Goodyera* and confirmed the same topology with aligned 79 plastid protein-coding genes (Supplementary Figure S4).

### Phylogenomic Analysis

**Figure 3:**
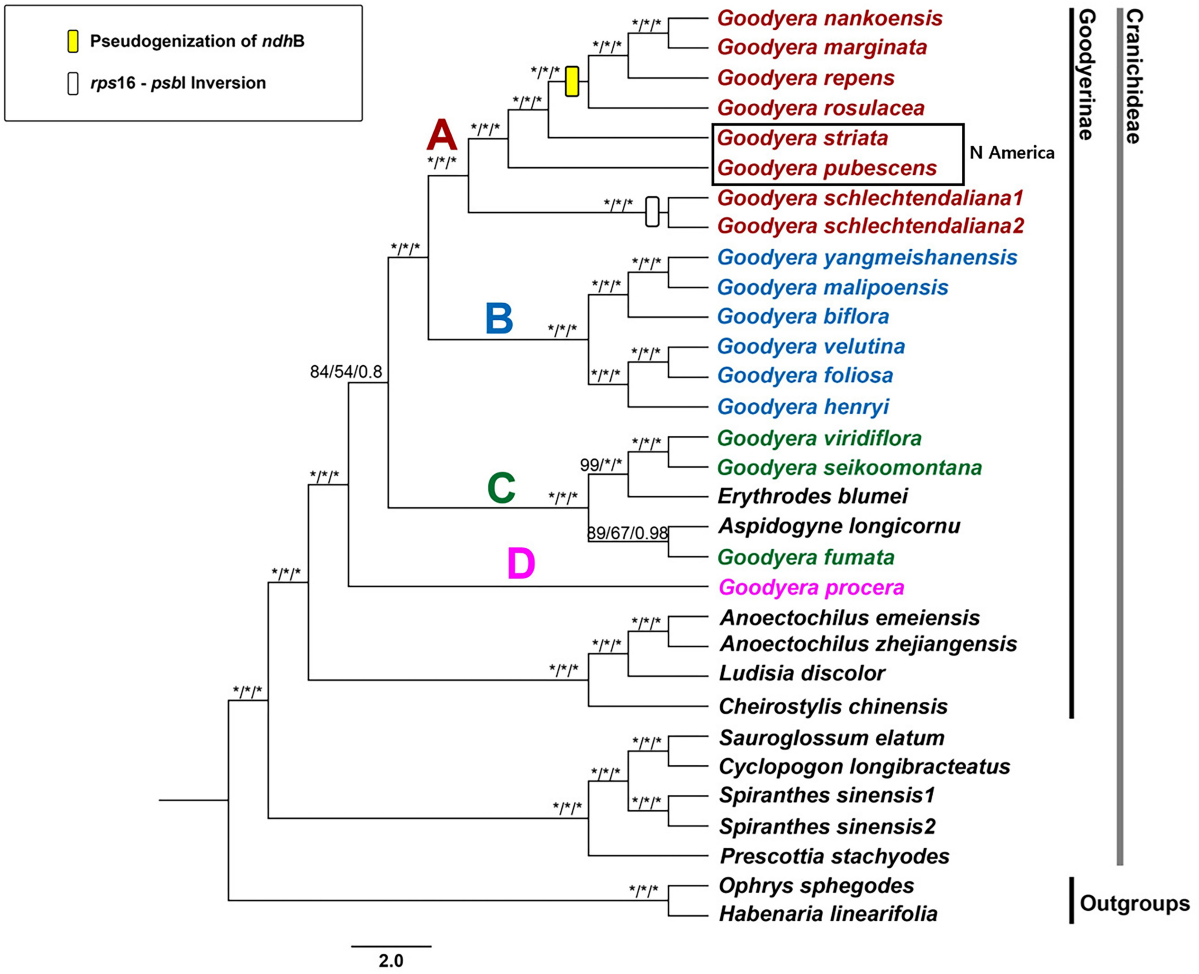
Strict consensus tree from the MP analysis of 31 orchids inferred from 79 plastid protein-coding genes. Numbers indicate support [maximum parsimony bootstrap (PBP)/maximum likelihood bootstrap (MBP)/posterior probability (PP)]. An asterisk (*) indicates that the node has 100% bootstrap or 1.00 posterior probability. The rectangle on the branch indicates the features of the plastome structure.

The phylogenetic trees were conducted by maximum parsimony (MP), maximum likelihood (ML), and Bayesian inference (BI) analyses with the data of the concatenated 79 plastid protein-coding genes (71,613 bp). The MP, ML, and BI trees shared the same topology and showed high supports [100% bootstrap (PBP, MBP) values and 1.00 Bayesian posterior probabilities (PP)] in most clades (Figure 3). The genus *Goodyera* was polyphyletic and subdivided into four subclades A, B, C, and D. *Erythrodes blumei* was a sister to *G. viridiflora* and *G. seikoomontana* (PBP = 99, MBP = 100, PP = 1), and *Aspidogyne longicornu* was a sister to *G. fumata* (PBP = 89, MBP = 67, PP = 0.98) (Figure 3). *G. procera* was a sister to the remaining species of *Goodyera* (Figure 3). Clade C was shown to be sister to clades A and B with relatively weak support values in all analyses (PBP = 84, MBP = 54, PP = 0.80). Clades A and B formed monophyly with strongly support values (PBP = 100, MBP = 100, PP = 1) (Figure 3).

### Divergence Time Estimation

**Figure 4:**
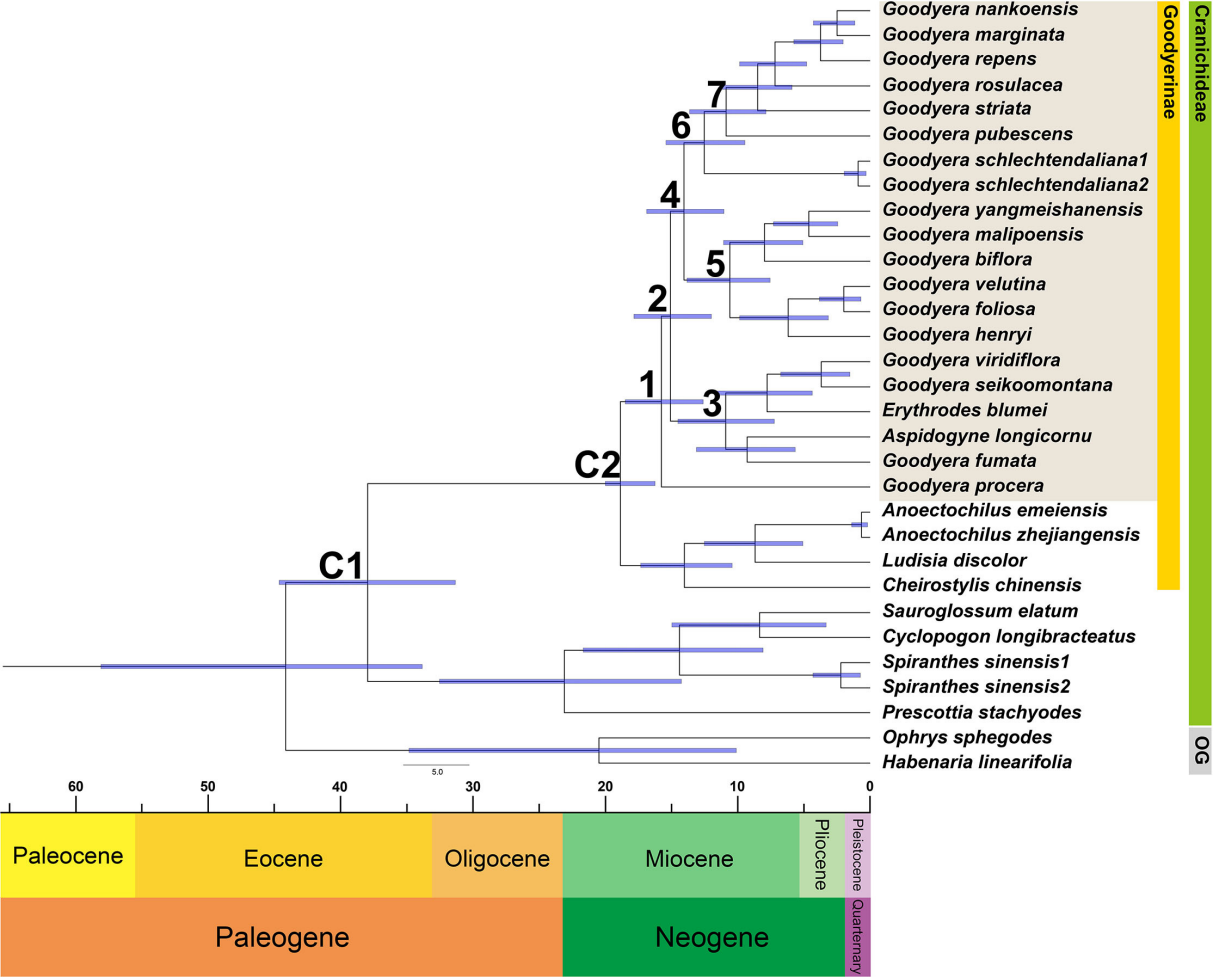
Chronogram showing the divergence times estimated in BEAST based on the combined 14 plastid DNA regions. The divergence times of the clades and subclades are shown near each node. Numbers 1–7 indicate nodes of interest (refer to Table 3 for details). Nodes labeled C1 and C2 are the calibration points used in the analysis (for details, refer to the Section “Materials and Methods”). Blue bars represent 95% highest posterior density for the estimated mean dates.

Through nucleotide diversity analysis in this study, 14 genes were selected to create data matric for divergence time estimation. The divergence time of *Goodyera* was estimated at 15.75 Mya in the mid-Miocene with a 95% HPD range of 12.6–18.49 Mya (node 1; Figure 4 and Table 3). The divergence of *Goodyera* excluding *G. procera* was estimated at 15.08 Mya (95% HPD = 11.99–17.83; node 2). The age of clade C (node 3), which comprised of polyphyletic groups from the Indomalesian to East Asia, was estimated at 10.91 Mya (95% HPD = 7.22–14.5 Mya) in the mid-Miocene. Clades A and B were split at 14.05 Mya (11.04–16.88 Mya, 95% HPD; node 4). The age estimate for the crown node of clade B (node 5), which primarily comprised of East Asian species, was dated at 10.59 Mya with a 95% HPD range of 7.55–13.82 Mya. Clade A (node 6) comprised of *Goodyera* species distributed in diverse continents was estimated at 12.51 Mya (95% HPD = 9.46–15.4 Mya) in the Miocene. Notably, the node 7, which has the distribution of Nearctic region including *G. pubescens* and *G. striata*, was diverged at 10.88 Mya (95% HPD = 7.86–13.63 Mya).

**Table 3:** Posterior age distributions of major nodes of *Goodyera* with results of ancestral area reconstruction using BBM analysis.

	**Age estimate**		**Ancestral and reconstruction** ^**b**^
**Node** ^**a**^	**Mean (Mya)**	**95% HPD (Mya)**	**BBM (%)**
1	15.75	12.6–18.49	AB (66), ABC (24)
2	15.08	11.99–17.83	AB (72), B (17)
3	10.91	7.22–14.5	AB (80)
4	14.05	11.04–16.88	B (83), AB (16)
5	10.59	7.55–13.82	B (94)
6	12.51	9.46–15.4	B (93)
7	10.88	7.86–13.63	–

a *Node numbers and biogeographical codes correspond to those in Figures 4, 5*.

b *Ancestral areas for each node are represented with ≥10%*.

### Ancestral Area Reconstruction

The summary of the ancestral distributions at the node of *Goodyera* inferred by BBM is shown in Figure 5 and Table 3. The BBM analyses support that *Goodyera* was originated in Southeast Asia + East Asia (AB), followed by ABC with 66% and 24%, respectively (node 1 in Figure 5). Similar results of origin were obtained for node 2 with 72% and node 3 with 80%, although it included South American species (*A. longicornu*) and East Asian species. The BBM reconstruction provides that East Asia (B) is the ancestral range for the subclades (node 5 and 6) in node 4 including mainly East Asian and East + Southeastern Asian species. In node 6, which included North American species (*G. pubescens* and *G. striata*) and East Asian *Goodyera* species, the BBM analyses indicated that East Asia (B) is the ancestral area for the subclades.

**Figure 5:**
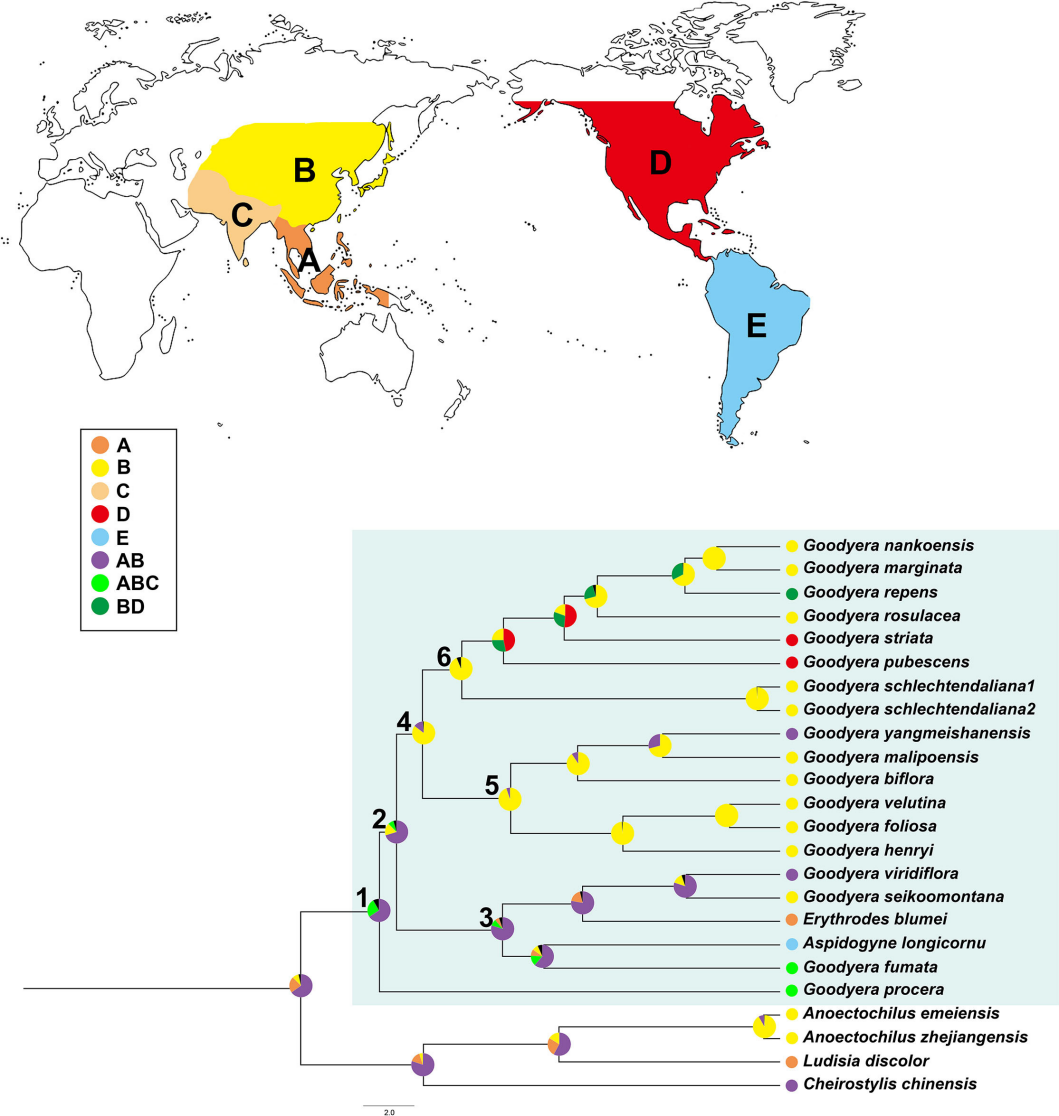
Summary of Bayesian binary method (BBM) model of ancestral area reconstruction in *Goodyera* based on a reduced BEAST combined-gene chronogram. The BBM ancestral area reconstructions with the highest likelihood are shown as pes for each clade of *Goodyera*. Biogeographical regions are used in BBM: A, Southeast Asia; B, East Asia; C, South Asia; D, North America; E, South America. Blue box = *Goodyera* clade. Numbers 1–6 indicate nodes of interest (for details, refer to Table 3).

## Discussion

### Characteristics of Plastid Genome in Genus *Goodyera*

Plastid genomes of *Goodyera* species have a quadripartite structure consisting of LSC and small single copy (SSC) regions separated by two IR regions (Figure 1). In this study, seven new plastid genomes of the genus *Goodyera* were completed, and the plastome sizes were 152,417 (*G. pubescens*) to 154,375 bp (*G. biflora*) with 37.2% of average GC content (Table 1). In monocot plastomes, unusual structural features happen frequently and provide informative infrageneric relationships. For example, Cyanotinae (Tradescantieae; Commelinaceae) had two large inversions in their plastid genomes, and its characteristics separated the other subtribes (Jung et al., 2021). In orchids such as *Cypripedium formosanum* and *Uncifera acuminata*, inversions of the plastid genome were verified (Lin et al., 2015; Liu et al., 2020). In this study, *G. schlechtendaliana* had one inversion (*rps*16—*psb*I), which is the unique feature found in the species (Supplementary Figure S2), and this result corresponds with that of Tu et al. (2021). We propose that AT-rich content of this inversion, which had weak hydrogen bonding, caused structural variations. We also confirmed that inversion sites had a larger proportion of AT contents compared to the average AT content of the LSC region. The GC content value in the LSC region is about 35% (Table 1), and its value in the region where the inversion occurs is about 20%. The IRs dividing LSC and SSC regions have highly conserved structures compared to the single-copy regions (Moore et al., 2007). We identified an IR expansion in members of *Goodyera* together with *Aspidogyne* and *Erythrodes*, but there is no difference in the boundaries of the quadripartite structure (Supplementary Figure S3).

Within *Goodyera* plastid genomes, four species (*G. nankoensis, G. marginata, G. repens*, and *G. rosulacea*) had a pseudogenized *ndh*B in plastid genomes. The pseudogenization or loss of *ndh* genes encoding the NAD(P)H-dehydrogenase complex has been found in many Orchidaceae species due to functional copies in the other genomes or redundant. For example, Apostasioideae have intact *ndh* genes whereas Vanilloideae lost *ndh* genes. However, some *ndh* genes are intact or lost in Cypripedioideae, Orchidoideae, and Epidendroideae (Wicke et al., 2011; Kim et al., 2015; Lin et al., 2015; Ruhlman et al., 2015; Liu et al., 2020; Tu et al., 2021). We identified that pseudogenization of *ndh*B is associated with a specific character of a clade including *G. nankoensis, G. marginata, G. repens*, and *G. rosulacea*, which is a potential molecular feature to distinguish members of *Goodyera* (Figure 3).

To identify the genetic divergence, the nucleotide diversity of CDS, tRNA, and rRNA was calculated among 18 *Goodyera* plastomes. Due to a highly conserved structure, the IR regions had lower values in nucleotide diversity than single-copy regions (Figure 2). We found a phylogenetically informative CDS dataset composed of 14 genes, which has high values (Pi > 0.013), and over 500 bp length and resulted in the same topology with a 79-aligned CDS dataset with high support value (Supplementary Figure S4).

### Phylogenomic and Phylogenetic Relationship Within Genus *Goodyera*

In contrast to the past phylogeny based on morphological characters, the phylogenetic relationships confirmed the monophyly of *Goodyera* based on molecular data including plastid, mitochondrial, and nuclear genes since 2000s. Hu et al. (2016) conducted the phylogenetic analysis of *Goodyera* using plastid and nuclear ribosomal DNA sequences and suggested the division into four sections of *Goodyera* associated with morphological characters and their distributions. Tu et al. (2021) utilized the whole plastid genome to study the phylogeny of Goodyerinae, especially *Cheirostylis* and *Goodyera* clades, and analyzed the plastome structure of Goodyerinae. In this study, we analyzed North American species based on whole plastid protein-coding genes and identified their relationships within *Goodyera*. In addition, we suggested the possibility that genome structure might be the molecular characters.

We obtained phylogenetic relationships of *Goodyera* including eight new plastid genomes with two species (*G. pubescens* and *G. striata*) distributed in the Nearctic region. Most species of *Goodyera* were affiliated to high-supported clades except for some clades including the clade C. The genus *Goodyera* was largely divided into four sections based on the 79 plastid-coding genes (Figure 3).

Clade D is composed of only one species, *G. procera*, which was a sister to the remainder of *Goodyera* with high support value (PBP = 100, MBP = 100, PP = 1 in Figure 3), which was corresponded with Tu et al. (2021). Hu et al. (2016) identified that *G. procera* formed a clade with high support, which has distinctive characters such as narrowly ovate-elliptic leaves or densely non-secund flowers. The position of *G. procera* is ambiguous to determine due to insufficient species of *Goodyera*, although that species was identified as the independent clade in this study.

Clade C has been shown as the non-monophyletic clade and a taxonomically debatable group. *Erythrodes* was affiliated to clade C with the characteristics of no-marking leaves and reflexed lateral sepals based on nuclear ITS, plastid *trn*L-F, and *mat*K genes (Hu et al., 2016). Chen et al. (2019) and Smidt et al. (2021) defined that clade C is the *Microchilus* subclade, in which diverse species exist including some *Goodyera* species. The results of Tu et al. (2021) showed that *Aspidogyne* was a sister to the rest of clade C, followed by *G. fumata*, whereas *Erythrodes* and *Goodyera* formed a subclade based on plastid-coding genes' dataset. Similarly, our study revealed that *Aspidogyne longicornu* was a sister to *G. fumata* and *Erythrodes blumei* was a sister to *G. viridiflora* and *G. seikoomontana*, respectively (Figure 3). Smidt et al. (2021) proposed the combinations of *Microchilus s.l*. including *Aspidogyne, Microchilus*, and *Kreodanthus* based on the results using ITS and *mat*K. However, the 79 CDS datasets revealed the placement of *Aspidogyne* within *Goodyera* clade. Therefore, more research should be done to clarify the phylogenetic relationships of those species.

Clade B included six species of *Goodyera* with strong support value (PBP = 100, MBP = 100, PP = 1) for all nodes in this study (Figure 3). Hu et al. (2016) described this clade as the silver or gold veins and not opened lateral sepals. They referred that *G. foliosa* formed two clades with *G. velutina* and *G. henryi* with high support values, respectively (Hu et al., 2016). In our study, we resolved the complex relationships among them that *G. velutina* and *G. foliosa* composed a high-supported clade and *G. henryi* was a sister to them (Figure 3).

Tu et al. (2021) suggested the combination of clades A and B into one clade; however, Hu et al. (2016) defined them as two sections, sect. *Goodyera* and sect. *Reticulum*, which was applied in this study. In our analysis based on 79 aligned plastid-coding genes, clade A included seven species, in which the upper taxon (*G. rosulacea, G. repens, G. marginata*, and *G. nankoensis*) has pseudogenized *ndh*B, which can be used as a synapomorphic feature (Figure 3). Additionally, they have a common character of glabrous inside the labellum, except for *G. schlechtendaliana* (Hu et al., 2016). All nodes had a strong support value compared to the results of Hu et al. (2016) or Chen et al. (2009).

In this study, a phylogenetic tree based on 79 plastid-coding genes provided phylogenetic relationships of 18 *Goodyera* species and improved their systematic positions, which were ambiguous in the previous studies. However, to understand the phylogenetic relationships of *Goodyera* species, more samples of *Goodyera* are required.

### Divergence Time and Biogeographical Origin

Overall, global warming was continued during the Miocene in comparison with the present day. In the mid-Miocene, the temperature was 4–5°C higher than now due to the influence of Mid-Miocene Climatic Optimum (MCO; *c*. 17 Mya), the global mean surface temperature during peak Miocene warmth (You et al., 2009; Hinsinger et al., 2013; Lawrence et al., 2021). Afterward, a decline in temperature, which is followed from mid- and late Miocene (*c*. 13 Mya) to Pliocene, triggered an expansion of temperature deciduous trees, grasses, composites, and other herbaceous dicots (Briggs, 1995; Lawrence et al., 2021). There are two situations of angiosperm floras by cooling, drying, and enhanced seasonability in the mid-latitudes; (1) deciduous trees and shrubs became dominant, and (2) monocots and dicots, mainly annual herbs, evolved and diverged (Briggs, 1995; Strömberg, 2011).

To date, some studies have been conducted on the biogeography of *Goodyera*. Smidt et al. (2021) conducted molecular dating and biogeographical analyses of Goodyerinae based on nuclear ribosomal ITS and plastid *mat*K sequences. Thiv et al. (2021) identified the phylogenetic location of *G. macrophylla*, which is the sister group to North American species although it is endemic species of Madeira. However, both papers are less reliable in that they used a small number of genes or introns when analyzing them.

Our molecular dating analyses suggest that the genus *Goodyera* was divaricated in the mid-Miocene (15.75 Mya, ±12.6–18.49) based on 14 plastid protein-coding genes (Figure 4 and Table 3), which was similar to the report of Smidt et al. (2021). Additionally, it originated in East + Southeast Asia, tropical or subtropical Asia including Indomalaya realm (Figure 5 and Table 3). *G. procera* (forming clade D) and clade C including other genera are distributed in subtropical or tropical Asia except for *A. longicornu* in South America and diverged *c*. 15.75 Mya and *c*. 10.91 Mya, respectively (Figure 4). We expect their most recent common ancestor (MRCA), which was distributed in a tropical or subtropical area and migrated to temperate Asia to survive against the rising temperature due to MCO. Smidt et al. (2021) proposed the Neotropical clade including *Aspidogyne* in Goodyerinae diverged *c*. 11 Mya and derived from Indomalayan ancestors, but our study lacked the Neotropical species in Goodyerinae and focused on the genus *Goodyera*, so further research is needed. Clades A and B ranged from tropical Asia to East Asia as discussed by Hu et al. (2016), diverged *c*. 12.51 Mya and *c*. 10.59 Mya at mid-Miocene, respectively (Figure 4). Specifically, clade A included North American species (*G. striata* and *G. pubescens*) and they diverged at 10.88 Mya (95% HPD = 7.86–13.63 Mya) in comparison with *c*. 8.4 Mya of Smidt et al. (2021) (Figure 4). The diversification of *Goodyera* mainly occurred in 14 Mya, when the temperature began to decrease, which is similar to the result of Briggs (1995). Although there are no significant differences between the two studies, the gaps are likely to happen as the result of the different calibration points or the number of genes. Our dataset provided a reliable value of the genus because 14 phylogenetically informative coding genes of plastid were used, compared to the use of a single *mat*K and ITS by Smidt et al. (2021). Consequently, it is conjectured that the MRCA of *Goodyera* species migrated from tropical Asia to temperate Asia due to MCO and divided into different lineages as the temperature began to decrease during Miocene.

### Intercontinental Disjunctions Between East Asia and North America in Genus *Goodyera*

Many researches about the biogeographical origin of diverse plants between East Asia and North America have been carried out based on molecular data (Donoghue et al., 2001; Wen et al., 2010, 2016; Kim et al., 2017, 2019). Wen et al. (2010) found that most temperate lineages had a dominance of directionality from East Asia to the North America by identifying 98 lineages with disjunct distribution between two regions: more than half of the lineages were confirmed to migrate from the Old World to the New World. In Cenozoic Era, there is a land bridge connecting continents, which is a migration route of Northern Hemisphere flora: one is the Bering Land Bridge (BLB) and the other is the North Atlantic Land bridge (NALB) (Wen et al., 2010; Deng et al., 2015, 2018; Kim et al., 2019). During the early Paleogene, the NALB, which extended across North America and Greenland to North-eastern Europe, is known as the main migration route for tropical taxa (Tiffney, 1985a; Tiffney and Manchester, 2001). On the other hand, the BLB, from Paleocene to Miocene, is considered as a connection between Asia and North America, which is a crucial route for the extension of temperate taxa (Tiffney, 1985b; Gladenkov et al., 2002).

Our BBM reconstruction suggests East + Southeast Asia as the ancestral area of *Goodyera* (Figure 5 and Table 3). Therefore, we suggest *Goodyera* most likely migrated from East Asia to North America via the BLB in the Miocene (Figure 5). Their MRCA remained in East Asia although several species were transferred to North America and then occurred the speciation in each location.

## Conclusion

Our study provides eight newly assembled plastid genomes of *Goodyera* R. Br. Additionally, an evolutionary framework to evaluate the systematic and biogeographical history of *Goodyera* was conducted. Unlike the previous studies, which mainly focused on species distributed in East Asia, this study conducted a phylogenomic study for the first time including North American species as well. Based on the plastid sequences, two abnormal structures of the plastid genomes in *Goodyera* have been revealed: an inversion in the LSC region of *G. schlechtendaliana* and a pseudogenization of *ndh*B gene in *G. nankoensis, G. marginata, G. repens*, and *G. rosulacea*. The phylogenetic analyses using the combined 79 protein-coding genes revealed that *Goodyera* is polyphyletic and can be divided into four sections with high support corresponding to some morphological characters. Furthermore, we resolved the phylogenetic relationships between unresolved species, suggesting further studies on the phylogeny of *Goodyera* by adding more samples and molecular data. Through the nucleotide diversity analysis, we found a phylogenetically informative dataset of 14 genes that resulted in same topology of phylogenetic tree to 79 plastid-coding datasets. Divergence time estimation and biogeographical analysis revealed that the common ancestors of all *Goodyera* species came from the tropical or subtropical Asia including Indomalaya realm in Miocene. In addition, we suggest East Asia as the major center of origin for taxa occurring in East Asia and North America, assuming the Bering Land Bridge played an important part in the migration in *Goodyera* between East Asia and North America. Our study added an example of the migration in the biogeographical history of the Northern Hemisphere.

## Data Availability Statement

The datasets presented in this study can be found in online repositories. The names of the repository/repositories and accession number(s) can be found at: National Center for Biotechnology Information (NCBI) BioProject database under accession numbers OM314910 - OM314917.

## Author Contributions

J-HK conceived and designed the experiments and revised the draft. J-HK and T-HK collected the plant materials. T-HK performed the experiments, analyzed the data, and wrote the draft. All authors agreed on the contents of the manuscript. All authors contributed to the article and approved the submitted version.

## Funding

This work was supported by the Gachon University research fund of 2019 (GCU-2019-0821) and the National Research Foundation of Korea (NRF) Grant Fund (NRF-2017R1D1A1B06029326).

## Conflict of Interest

The authors declare that the research was conducted in the absence of any commercial or financial relationships that could be construed as a potential conflict of interest.

## Publisher's Note

All claims expressed in this article are solely those of the authors and do not necessarily represent those of their affiliated organizations, or those of the publisher, the editors and the reviewers. Any product that may be evaluated in this article, or claim that may be made by its manufacturer, is not guaranteed or endorsed by the publisher.

## Supplementary Material

Supplementary Figure S1 Complete plastid genome maps of *Goodyera* species and *Spiranthes sinensis*. Genes shown inside the circle are transcribed clockwise, and outside are counterclockwise. Genes belonging to different functional groups are colored. The inner circle shows the LSC, SSC, and IR regions. The darker gray in the inner circle corresponds to GC content, and the lighter gray corresponds to AT content.

Supplementary Figure S2 The alignment of plastid genome sequences based on mVISTA. Plots of percent sequence identity of the plastid genomes of 20 *Goodyera* clade species with *Anoectochilus emeiensis* as a reference. The percentage of sequence identities was estimated, and the plots were visualized in mVISTA. CDS means coding sequences and CNS means non-coding sequences.

Supplementary Figure S3 Comparisons of LSC, SSC, and IR regions boundaries between 20 species of *Goodyera* clade.

Supplementary Figure S4 The maximum likelihood tree of 31 orchids inferred from 14 plastid protein-coding gene datasets. Numbers indicate support [maximum parsimony bootstrap (PBP)/maximum likelihood bootstrap (MBP)]. An asterisk (^*^) indicates the node has 100% bootstrap.

Supplementary Table S1 List of samples for total DNA extraction and assembly information.

Supplementary Table S2 Nucleotide diversity (Pi) of 18 *Goodyera* species.
