# Supplementary figures and images for: Molecular Phylogeny and Historical Biogeography of Goodyera R. Br. (Orchidaceae): A Case of the Vicariance Between East Asia and North America
Source: Front Plant Sci. 2022 May 2;13:850170. doi: 10.3389/fpls.2022.850170 (PMC9108766; doi:10.3389/fpls.2022.850170)

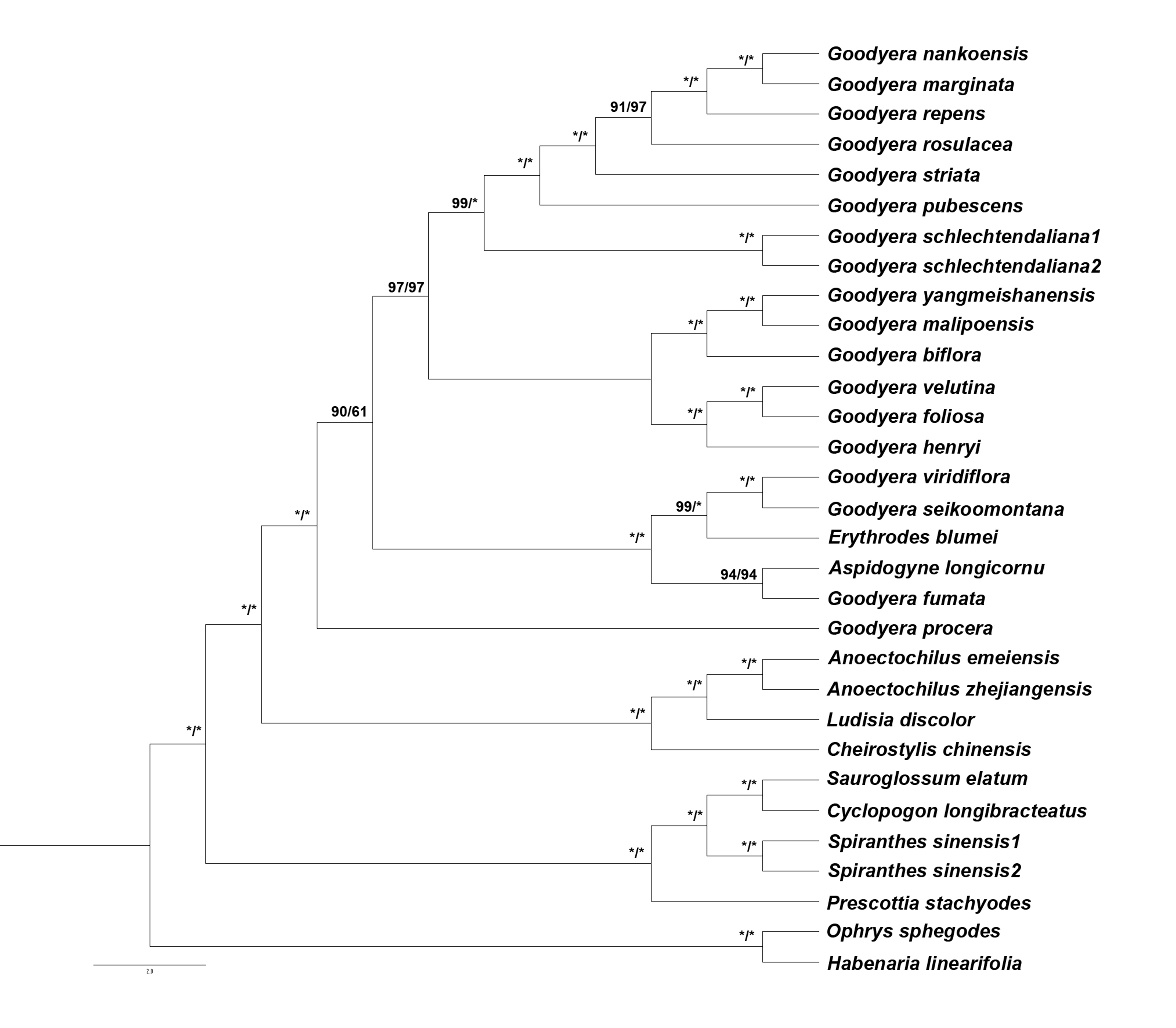

Supplement: Supplementary Figure S4 — The maximum likelihood tree of 31 orchids inferred from 14 plastid protein-coding gene datasets. Numbers indicate support [maximum parsimony bootstrap (PBP)/maximum likelihood bootstrap (MBP)]. An asterisk (*) indicates the node has 100% bootstrap. [file Image_4.jpg]
